# Supplementary material for: The long term impact of Daylight Saving Time regulations in daily life at several circles of latitude
Source: Sci Rep. 2019 Dec 5;9:18466. doi: 10.1038/s41598-019-54990-6 (PMC6895179; doi:10.1038/s41598-019-54990-6)
Supplement: Supplementary file 1 — Supplementary material to “The long term impact of Daylight Saving Time regulations in daily life at several circles of latitude” [file 41598_2019_54990_MOESM1_ESM.pdf]

Supplementary material to "The long term impact of Daylight Saving Time regulations in daily life at several circles of latitude"

José María Martín-Olalla*

Universidad de Sevilla. Facultad de Física. Departamento de Física de la Materia Condensada. ES41012 Sevilla. Spain

(Dated: July 9, 2019)

Supplementary material

* olalla@us.es; Twitter: @MartinOlalla_JM

Typeset by REVTEX

CONTENTS

Supplementary material	2
S.1. Mathematical framework	2
S.2. Geophysical framework	4
S.3. Figures	7
S.4. Tabular material	12

SUPPLEMENTARY MATERIAL

S.1. Mathematical framework

Every respondent of a time use survey filled a diary which consisted of $N_0 = 144$ time slots —each one representing ten minutes— or indexes. For every index, respondents indicated which activity was being performed, where and with whom. The sleep/wake cycle and the labour cycle —two of the most basic, universal human activities— will be studied after identifying in every diary activity codes corresponding to sleeping or working.

An activity will be represented by a state function A which can take only two values $a_1 = 1$, if the respondent is doing the activity at a given index, and $a_0 = 0$ if the respondent is not doing the activity. Therefore we deal with at two-state system.

The average value of A is called the daily rhythm R as it counts the shares of population doing the activity as a function of time. For a survey of size N if m individuals are doing the activity and $N - m$ are not doing the activity then:

$$R(i) = \frac{m(i) \times a_1 + (N - m(i)) \times a_0}{N} = \frac{1}{N} m(i). \tag{S1}$$

Daily rhythms can be computed for winter R_w and summer R_s and then seasonal differences are given by $\Delta R(i) = R_s(i) - R_w(i)$. It is very important of understand that this seasonal difference are taken at constant i, that is at a given local time. Taking into account summer time regulations seasonal differences are computed for universal times differing one whole hour: $R_s(t) - R_w(t + 1\,\mathrm{h})$.

If human activity were not seasonal then ΔR should fade to zero for large enough values of sample size. But with finite samples ΔR is experimentally non-zero and a test statistic is needed to sustain or reject the null hypothesis $H_0 : R_s(i) - R_w(i) = 0$, tested on every index i. The appropriate test in this case is the Welch's unequal variance t-test since R is an average value and its variance can be obtained easily as:

$$s(i)^2 = R(i) \cdot (1 - R(i)). \tag{S2}$$

The Welch's statistics normalises the raw difference of average values ΔR by sample size and sample variance with the following formula:

$$\Delta K_w(i) = \sqrt{\mathcal{N}(i)} \cdot \frac{\Delta R(i)}{s_{\mathrm{rms}}(i)\sqrt{2}} \tag{S3}$$

where $s_{\mathrm{rms}}(i)$ is the root mean square of the variances and $\mathcal{N}(i)$ is the harmonic average of sample sizes weighted by the variances:

$$\mathcal{N}(i) = \frac{s_s^2(i) + s_w^2(i)}{\dfrac{s_s^2(i)}{N_s} + \dfrac{s_w^2(i)}{N_w}} \tag{S4}$$

As a result ΔK_w can compare differences from surveys and groups differing in sample size. Finally evaluating the cumulative distribution function of the t-Student at $\Delta K_w(i)$ a p-value is obtained for the null hypothesis $H_0 : R_s(i) - R_w(i) = 0$. Notice that $p(i)$. The number of degrees of freedom for this computus is determined by the Welch-Satterthwaite equation:

$$\nu(i) = \frac{\left(N_w s_w^2(i) + N_s s_s^2(i)\right)^2}{N_w^2 s_w^4(i) + N_s^2 s_s^4(i)} \mathfrak{N}(i) \tag{S5}$$

where $\mathfrak{N}(i)$ is the harmonic average of the sample sizes weighted by Ns^2.

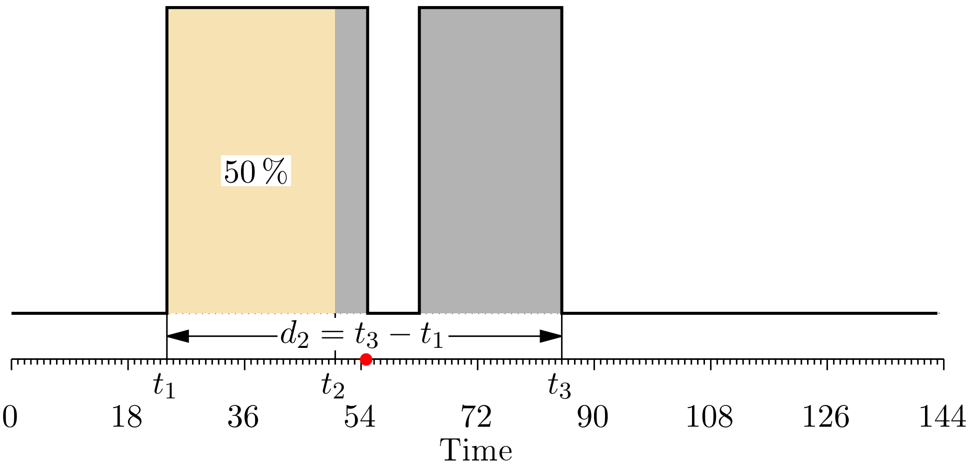

Figure S.1 A sketch of the state function for some activity and some individual. The function is activated (upper bound) or deactivated (lower bound). The shaded area computes the duration of the activity d_1: the first half is noted in a lighter ink; the second half, in a darker ink. Onset time t_1 is the first occurrence of the activity. Offset time t_3 is the last occurrence. The amplitude d_2 is the time distance from onset to offset. The center of gravity t_2 is the moment when half the duration has been consumed. The red circle indicates the midpoint from t_1 and t_3. If the activity were unimodal —no breaks— the midpoint and the center of gravity would match to each other. It is not the case in this sketch.

An alternative description of an activity can be obtained from five stochastic variables that result from analysing the state of an activity during one cycle for every individual. A paradigmatic state function A is sketched in Figure S.1. The shaded area is the duration of the activity computed just by adding up all contributions to the activity in one diary:

$$d_1 = \sum_{i=1}^{N_0} A(i). \tag{S6}$$

From this quantity the center of gravity t_2 can be determined as the moment when half duration has been consumed and half remains to be consumed.

The onset time t_1 can be defined as the first occurrence of the activity and the offset time t_3 as the last occurrence. The amplitude d_2 is the time distance from offset to onset.

It must be noted that *duration* is unequivocally computed. So is the *center of gravity*, up to the arbitrary starting point of the cycle. On the contrary t_1, t_2 and d_2 can only be identified with onset, offset, and amplitude as long as A is not too fragmented and, perhaps, as long as the respondent was actually sleeping by 4am. The activity of a tiny fraction of individuals part ways from this simple scheme, which may influence results on these variables.

On the other hand three of these quantities —offset, onset and the center of gravity, labelled with the letter t— are time-marks and will always be expressed local time, thus relative to a time zone. Therefore they are sensitive to time arrangements. The remaining two quantities —daily duration and amplitude, labelled with the letter d— are elapsed times and do not depend on time zones.

Every of these five quantities is a stochastic discrete variable with only $N_0 = 144$ possible outcomes. It can then be determined how many counts (respondents) $n(i)$ populate a given index i and the experimental (or sample) cumulative distribution function:

$$P(i) = \frac{1}{N} \sum_{k=1}^{i} n(k) \tag{S7}$$

These cumulative probabilities can be computed for the winter P_w and summer P_s partitions and the seasonal difference is just $\Delta P(i) = P_s(i) - P_w(i)$.

As for daily rhythms, a test is needed to assess the significance of seasonal deviations. However some differences must be noted. First here the null hypothesis $H_0 : P_s - P_w = 0$ inspects whether two experimental cumulative

distributions come from the same parent, unknown distribution. Therefore the null hypothesis is tested for one stochastic variable and it is the maximum deviation that carries the relevant information. Instead R is an average value only, and the test is run for every index i.

For $P_s - P_w$ the Kolmogorov-Smirnov test is the appropriate choice but due to the discrete nature of the variables no p-values can be obtained from the test. As an alternative a permutation test will be carried out for this purpose. The seasonal index —which links one respondent to one season— will be permuted or reshuffled M times. For every permutation two samples of size N_s and N_w will be extracted. The deviations of the permuted samples will be compared to the parent seasonal deviation in search of larger the deviations. The p-value of the test is the fraction $p = M_0/M$ of permutations with larger results than the seasonal parent sample.

Moreover, in this case tailed (one-sided) tests are meaningful since, unlike R, P is monotonic on the index i. Therefore the seasonal absolute deviation $D_0 = \max\{|P_s(i) - P_w(i)|\}$ and the signed deviations $D_0^+ = \max\{P_s(i) - P_w(i)\}$ and $D_0^- = \min\{P_s(i) - P_w(i)\}$ will be compared with the shuffled deviations to test the null hypotheses $H_0 : P_s - P_w = 0$, $H_0 : P_s - P_w \geq 0$ and $H_0 : P_s - P_w \leq 0$ against the alternates $H_1 : P_s - P_w \neq 0$, $H_1 : P_s - P_w < 0$ and $H_1 : P_s - P_w > 0$. Four possible outcomes are possible:

equal **(=):** if $D_j \geq D_0$ at least in 5 % of the permutations so that the null hypothesis $H_0 : P_s - P_w = 0$ sustains.

less **(<):** if $D_j^- < D_0^-$ in less than 5 % of the permutations and $D_j^+ \geq D_0^+$ at least in 5 % of the permutations so that the null hypothesis $H_0 : P_s - P_w \leq 0$ sustains and the null hypothesis $H_0 : P_s - P_w \geq 0$ is rejected. First-order stochastic dominance results in the summer distribution delaying relative to the winter distribution for some index.

greater **(>):** if $D_j^+ > D_0^+$ in less than 5 % of the permutations and $D_j^- \leq D_0^-$ at least in 5 % of the permutations so that the null hypothesis $H_0 : P_s - P_w \geq 0$ sustains and the null hypothesis $H_0 : P_s - P_w \leq 0$ is rejected. First-order stochastic dominance results in the summer distribution advancing relative to the winter distribution for some index.

not equal **(≠):** if both $D_j^+ > D_0^+$ and $D_j^- < D_0^-$ in less than 5 % of the permutations so that all the three null hypotheses are rejected and no stochastic dominance is indicated.

Permutation tests only require the knowledge of the raw seasonal difference $P_s - P_w$ but for comparing the size of deviations among surveys which differs in size, the Kolmogorov-Smirnov (KS) statistics or distance provides a normalised deviation. It is expressed as:

$$\Delta K_{ks}(i) = \sqrt{N_h} \cdot \Delta P(i) = \sqrt{N_h} \cdot (P_s(i) - P_w(i)), \tag{S8}$$

where N_h is the unweighted harmonic average of sample sizes:

$$N_h = \frac{2}{\dfrac{1}{N_w} + \dfrac{1}{N_s}}. \tag{S9}$$

The Kolmogorov-Smirnov cumulative distribution function evaluated at $\max|\Delta K_{ks}|/\sqrt{2}$ provides p-values for the null hypothesis in the continuous case. Also in that case, the p-value in sided tests turns to be equal to $\exp\left\{-N_h \left(D_0^\pm\right)^2\right\}$. In this study these p-values will only be used as a reference for graphical purposes.

S.2. Geophysical framework

Figure S.2 shows a solar location diagram for latitude 40°. The thick blue circle is the local horizon. Around this circle local azimuths or bearings are displayed. Dashed blue circles display equidistant points to the center, which can be expressed as an angular distance —the zenith angle— taking into account Earth's radius. Numbered thick blue lines display mean solar time.

The map of the Earth provides a context as a background image. Accidentally it is centered at longitude −3°. Had the diagram been plotted for latitude 90°, the local horizon would have been the Equator, azimuths would have been meridians and zenith lines would have been circles of latitude.

It is daytime in the center and an observer located on its zenith sees it in the light hemisphere of the Earth whenever the subsolar point —the point with the Sun overhead, which apparently moves as Earth rotates— lies inside the thick blue circle. Otherwise, the observer could not the Sun and would see the center in the dark hemisphere of the Earth.

Whenever the subsolar point touches the thick blue circle the Sun is rising or setting in the center, and the observer would be seeing the line of umbra crossing the center. Solar zenith angle is invariably 90° and solar altitude is invariably 0°.

The subsolar point is confined year round to the tropical range. Therefore the intersect of the local horizon with the Tropics defines the locus of sunrises and sunsets which extends "horizontally" over a range of azimuths. Figure S.2 shows the limiting conditions —winter sunrise and sunset, summer sunrise and sunset— as light blue points with labels. The spread in azimuth is the angle subtended by the center and the summer sunrise and winter sunrise points, noted by a thin blue line. The spread of sunrise/sunset times is defined by the spherical angle subtended by the pole and rise points, shown in the figure by dashed, gray arcs. This angle can be converted into time taking into account Earth's angular speed of rotation $\Omega = 15\,^\circ\,\mathrm{h}^{-1}$ and can be projected into a clock with 24-H analog dial as in Figure 1.

Noon occurs invariably when the subsolar point intersects the local meridian or the local anti-meridian. The subsolar point reaches the daily, shortest distance to the center and the lowest zenith angle. In the figure winter noon and summer noon are noted by orange dots. In the extra-tropical range the azimuth is invariable but the zenith angle scores $\theta_s = |\phi| - \varepsilon$ in summer and $\theta_w = |\phi| + \varepsilon$ in winter. The "vertical" spread invariably amounts to $\theta_s - \theta_w = 2\varepsilon = 47^\circ$. Notwithstanding all this, the cosine of the solar zenith angle is a more sensitive quantity to trace since it expresses the efficiency of solar insolation as a fraction of the insolation measured at the subsolar point, where the Sun is overhead, the zenith angle is zero and no shadow is cast.

As a summary of seasonal changes, the intersect of winter sunrise meridian with the Tropic of Cancer (summer solstice line) occurs at zenith angle $\sim 60^\circ$. That is at the 40th circle of latitude, by the time of winter sunrise, the Sun has already climbed up to 30° —$\cos\theta_s = 50\,\%$— above the horizon in summer.

If the diagram is plotted for a higher latitude, orange dots signalling solar zenith angle at noon will come closer to the horizon, the spread will still be 2ε but values would differ, altering also $\cos\theta$. Light blue dots will open towards the local anti-meridian (summer) and will close towards the local meridian (winter), enlarging the spread of horizontal variations.

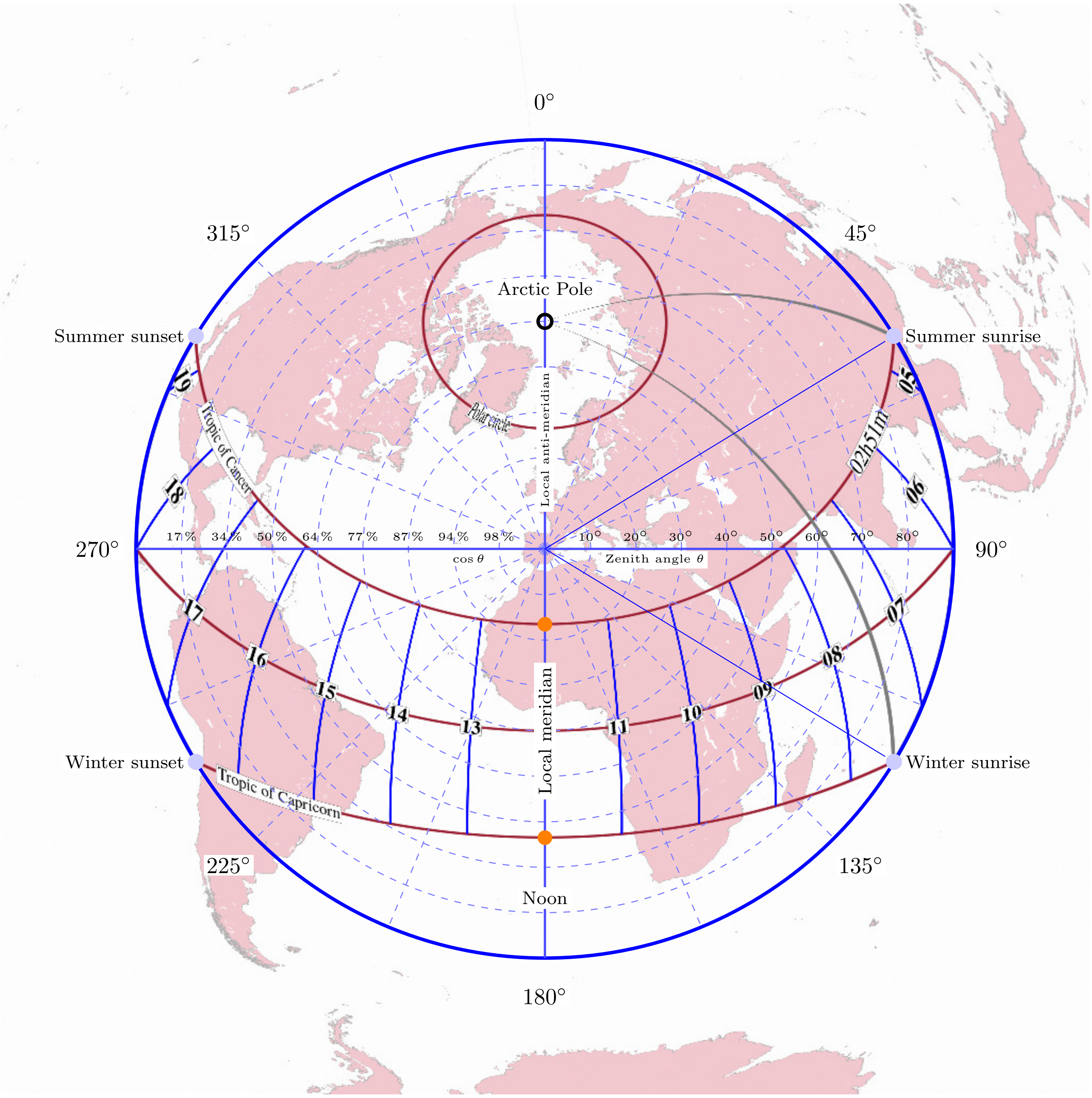

Figure S.2 The Earth as seen from an observer located in the zenith of the Iberian peninsula (the center, longitude $-3.0°$, latitude $40°$). The compass rose signals azimuths, great circles leaving the location on every direction, alike meridians in the Pole. Dashed blue circles display distance to the center, converted into angle —zenith angle— after scaling by Earth's radius, alike parallels in the Pole. The thick blue line is the local horizon, alike the Equator for the Poles. It locates $90°$ from the center —or $\sim 10\,000$ km as per the original definition of meter—. Beyond this line nothing can be viewed from the observer —though Earth boundaries are shown in the picture— as it lies in the opposite hemisphere of the center. As Earth revolves around the Sun and rotates around the Pole the subsolar point —where the Sun is overhead— transits the Tropic of Cancer (by the June solstice), the Equator (at the equinoctes) and the Tropic of Capricorn (by the December solstice). Every day it crosses the $90°$ circle twice. Whenever this happens the Sun is up the horizon in the center and the observer sees it in the line of umbra light hemisphere of the Earth. Whenever the subsolar point transits the local meridian it is noon, the subsolar point is in its daily shortest range to the center, the solar zenith angle is the smallest and solar altitude, the highest. Six ephemerides are noted: (1) orange dots punctuate winter and summer noon, they account for the zenith or "vertical" seasonal variation; (2) light blue dots punctuate the subsolar point at winter and summer sunrises and sunsets, they carry the azimuth or "horizontal" variation, noted by the thin, solid blue lines on the center. Numbered thick solid lines display means solar time, they are great circles passing through the Poles. The gray great circles that join subsolar point at summer sunrise and at winter sunrise to the Pole punctuate the spread of sunrise times, which is annotated over the Tropic line. Notice that in summer, at the time of the winter sunrise, the center is seeing the Sun some $60°$ from the zenith or $30°$ up the horizon. The setting is not altered if the longitude of the center is altered, save for the map of the Earth in the background. Instead, if latitude increases then the orange dots move downwards, summer sunrise/sunset opens towards the local anti-meridian and winter sunrise/sunset closes towards the local meridian. The opposite occurs if latitude decreases. Boundary lines and shapes were taken from https://www.naturalearthdata.com/. A solar location diagram for latitude $51°30'$ is on display at the United Kingdom Hydrographic Office http://astro.ukho.gov.uk/nao/services/ais58.pdf.

S.3. Figures

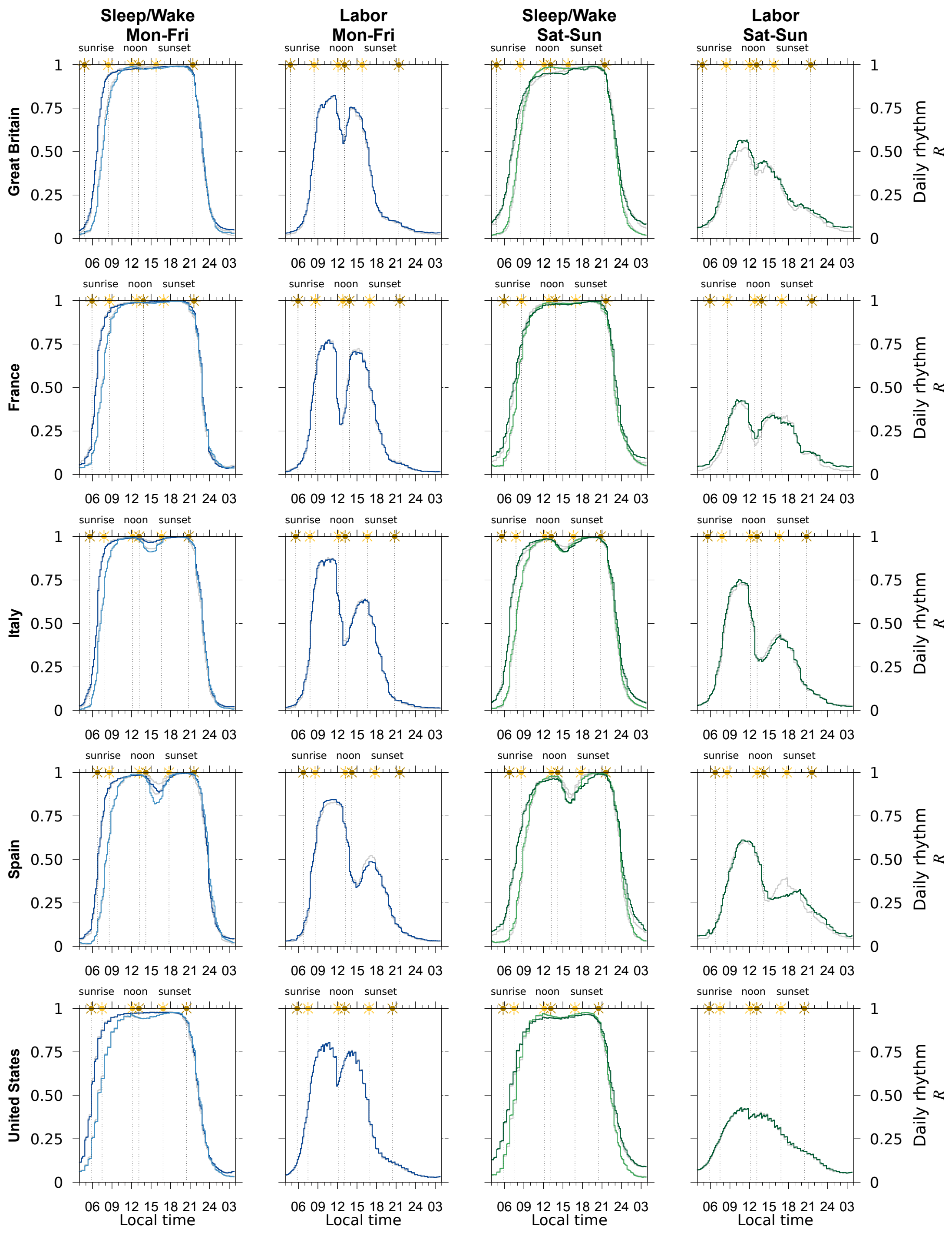

Figure S.3 The sleep/wake and labour daily rhythms in winter and summer. Blueish inks display week-day rhythms (groups 1 and 2). Greenish inks display week-end rhythms (groups 3 and 4). Darker lines apply to workers (groups 1 and 3) in summer, lighter lines apply to non-workers (groups 2 and 4) in summer. Even lighter lines, hard to visualise, display winter daily rhythms. Vertical lines highlight solar events —sunrise, noon and sunset— obtained for the population weighted median latitude and longitude of each country.

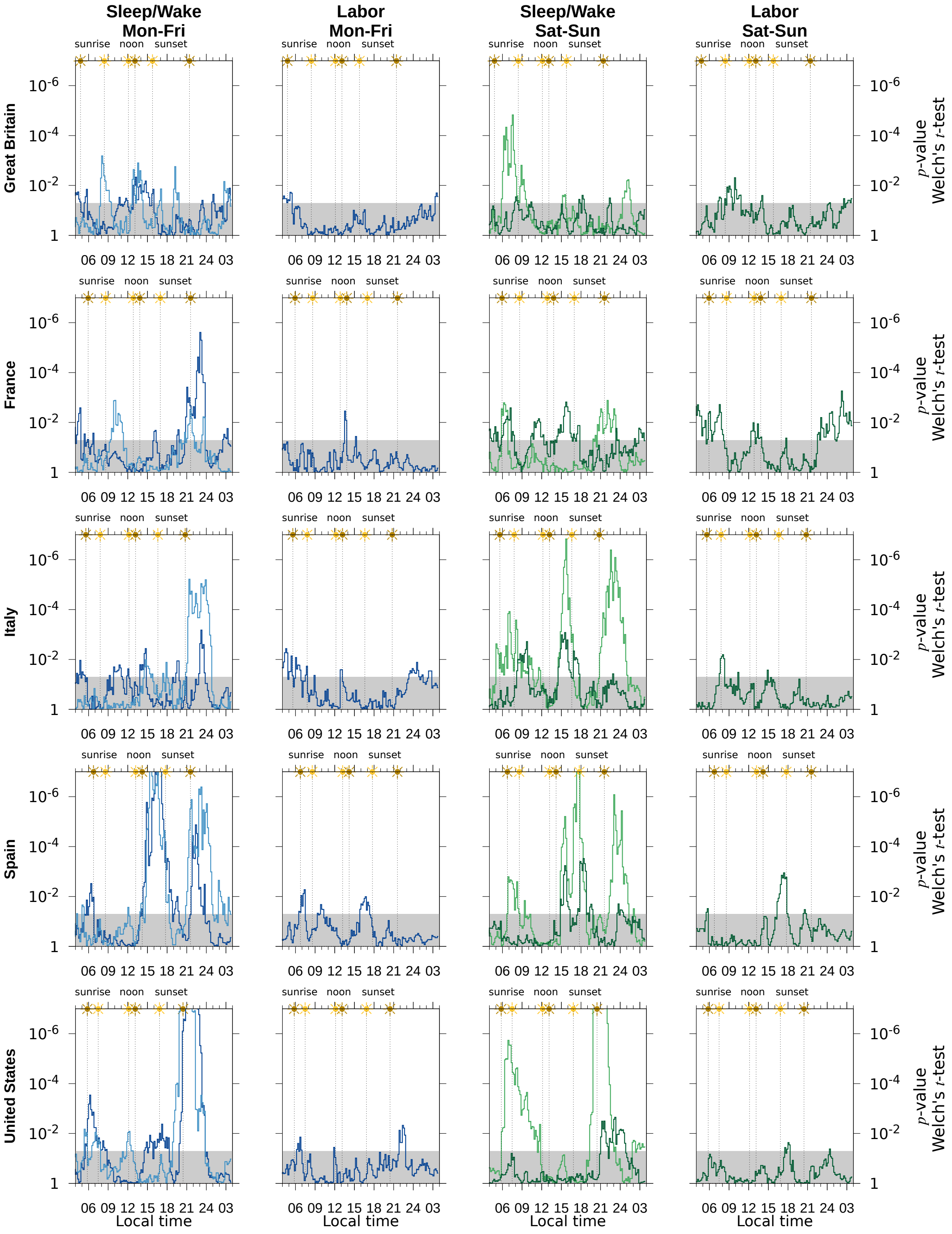

Figure S.4 Probabilistic values obtained from the Welch's t-test on daily rhythms (see Eqs (S3) to (S5)). The gray band highlights the region $p(i) > \alpha$ where the null hypothesis $H_0 : R_s(i) - R_w(i) = 0$ would sustain at the standard level of significance $\alpha = 0.05 = 10^{-1.30}$. Vertical lines show solar ephemerides (sunrise, noon and sunset) in winter and summer as measured in local time. Darker sun applies to summer. Colour lines follow Figure S.3.

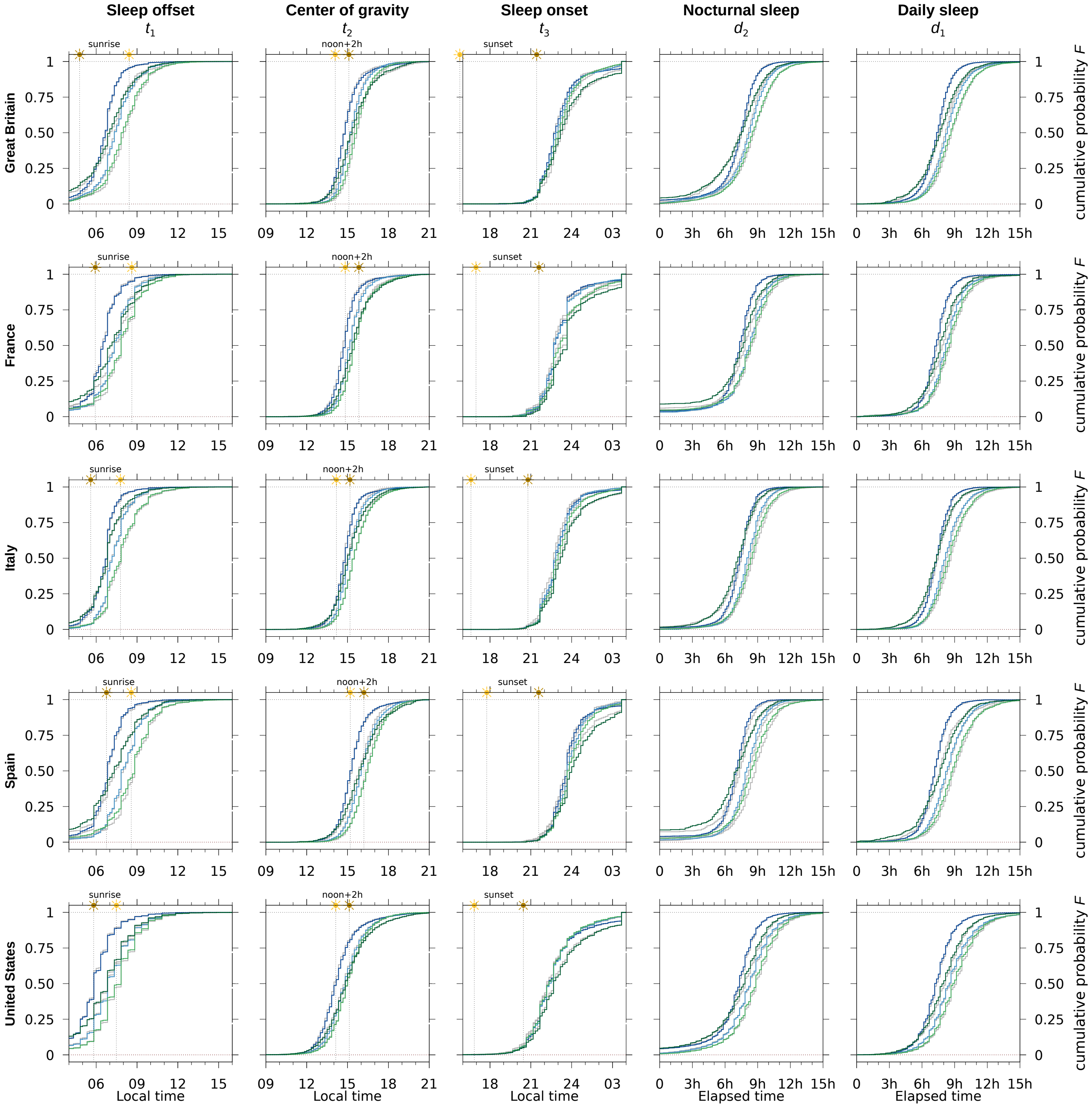

Figure S.5 The sample cumulative distribution function of the sleep/wake stochastic variables (columns) for different countries (rows) and seasons: thicker lines display summer values, thinner lines (hard to visualise) display winter values. Blueish inks display groups 1 and 2 (week days) and greenish inks, groups 3 and 4 (week ends). Vertical lines display sunrise, noon and sunset times as measured local time.

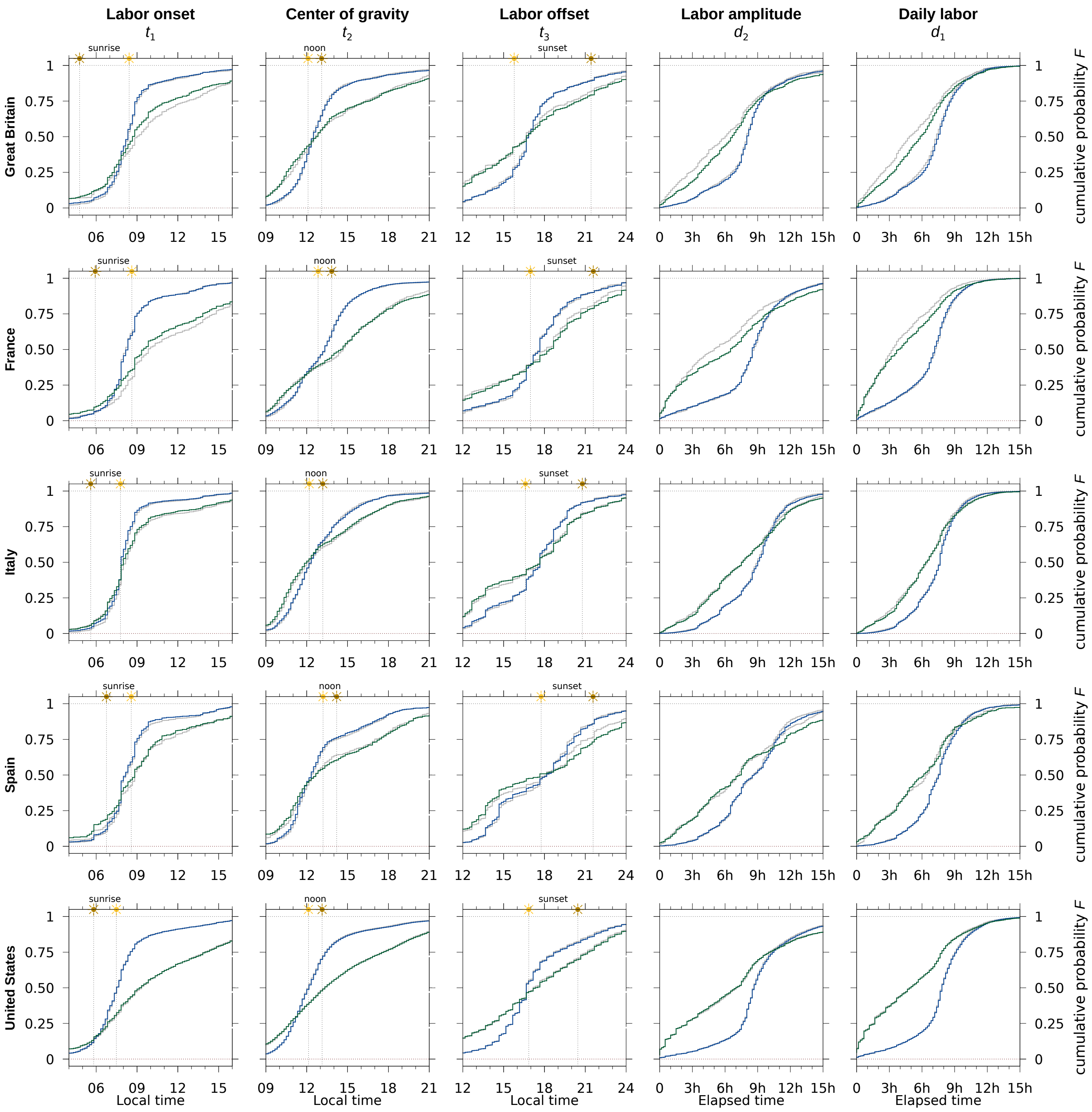

Figure S.6 Same as Figure S.5 but for the labour stochastic variables.

S.4. Tabular material

KS statistics		Group 1<br>Mon-Fri<br>employees			Group 2<br>Mon-Fri<br>non-employees			Group 3<br>Sat-Sun<br>non-employees			Group 4<br>Sat-Sun<br>employees	
	t_m	$P_s \leftrightarrow P_w$	p	t_m	$P_s \leftrightarrow P_w$	p	t_m	$P_s \leftrightarrow P_w$	p	t_m	$P_s \leftrightarrow P_w$	p
**Great Britain**												
Sleep offset	05:50	$0.19 = 0.16$	$10^{-0.53}$	08:10	$0.72 < 0.77$	$10^{-2.69}$	08:10	$0.77 = 0.72$	$10^{-0.53}$	07:40	$0.38 > 0.33$	$10^{-2.92}$
Center of gravity	16:10	$0.89 = 0.91$	$10^{-0.46}$	16:00	$0.76 = 0.78$	$10^{-0.13}$	15:10	$0.44 > 0.36$	$10^{-2.23}$	15:40	$0.49 > 0.46$	$10^{-1.77}$
Sleep onset	23:20	$0.65 = 0.67$	$10^{-0.27}$	23:40	$0.70 = 0.68$	$10^{-0.17}$	22:30	$0.28 = 0.22$	$10^{-0.88}$	01:00	$0.90 = 0.87$	$10^{-0.54}$
Onset to offset	07h50m	$0.54 = 0.51$	$10^{-0.55}$	09h30m	$0.76 < 0.80$	$10^{-1.93}$	07h50m	$0.51 = 0.54$	$10^{-0.13}$	08h30m	$0.43 > 0.40$	$10^{-2.18}$
Sleep time	07h40m	$0.50 = 0.47$	$10^{-0.81}$	09h20m	$0.70 < 0.76$	$10^{-2.53}$	08h50m	$0.71 = 0.67$	$10^{-0.31}$	08h00m	$0.29 > 0.26$	$10^{-2.05}$
**France**												
Sleep offset	06:50	$0.57 < 0.59$	$10^{-3.34}$	07:40	$0.55 \neq 0.57$	$< 10^{-S}$	07:20	$0.52 > 0.45$	$10^{-2.47}$	06:30	$0.18 > 0.15$	$10^{-3.19}$
Center of gravity	14:50	$0.48 < 0.53$	$10^{-3.69}$	16:20	$0.86 = 0.89$	$10^{-0.56}$	17:20	$0.89 = 0.92$	$10^{-0.43}$	16:00	$0.67 = 0.68$	$10^{-0.22}$
Sleep onset	23:00	$0.55 < 0.60$	$10^{-7.00}$	23:30	$0.66 < 0.69$	$< 10^{-S}$	23:20	$0.44 = 0.49$	$10^{-0.88}$	23:00	$0.45 < 0.48$	$10^{-3.21}$
Onset to offset	07h20m	$0.41 > 0.37$	$10^{-4.55}$	07h10m	$0.22 \neq 0.20$	$10^{-6.15}$	08h10m	$0.61 > 0.53$	$10^{-2.57}$	09h10m	$0.68 > 0.64$	$10^{-3.40}$
Sleep time	07h20m	$0.42 > 0.38$	$10^{-2.36}$	07h30m	$0.24 = 0.22$	$10^{-0.56}$	08h00m	$0.54 > 0.47$	$10^{-2.38}$	09h00m	$0.62 > 0.58$	$10^{-2.44}$
**Italy**												
Sleep offset	05:50	$0.15 = 0.14$	$10^{-0.36}$	07:00	$0.41 = 0.43$	$10^{-0.21}$	08:30	$0.90 = 0.88$	$10^{-0.30}$	07:10	$0.34 > 0.31$	$10^{-2.72}$
Center of gravity	15:10	$0.66 < 0.69$	$10^{-1.61}$	15:20	$0.55 < 0.60$	$10^{-2.75}$	15:00	$0.45 = 0.47$	$10^{-0.10}$	16:10	$0.72 = 0.72$	$10^{-0.05}$
Sleep onset	23:10	$0.59 < 0.63$	$10^{-2.76}$	23:10	$0.60 < 0.66$	$10^{-4.77}$	22:50	$0.35 = 0.38$	$10^{-0.67}$	23:10	$0.53 < 0.57$	$10^{-5.02}$
Onset to offset	07h30m	$0.45 > 0.41$	$10^{-2.44}$	08h00m	$0.39 > 0.32$	$10^{-5.80}$	07h30m	$0.51 > 0.46$	$10^{-2.05}$	09h10m	$0.66 > 0.60$	$< 10^{-S}$
Sleep time	08h00m	$0.67 > 0.64$	$10^{-1.63}$	07h50m	$0.34 > 0.28$	$10^{-4.29}$	07h40m	$0.52 = 0.49$	$10^{-0.88}$	09h00m	$0.59 > 0.54$	$< 10^{-S}$
**Spain**												
Sleep offset	06:30	$0.33 > 0.28$	$10^{-1.94}$	07:50	$0.37 > 0.35$	$10^{-3.13}$	06:20	$0.29 > 0.24$	$10^{-4.68}$	08:20	$0.37 > 0.33$	$< 10^{-S}$
Center of gravity	15:20	$0.50 = 0.48$	$10^{-0.22}$	16:10	$0.53 < 0.57$	$10^{-2.23}$	14:50	$0.19 = 0.17$	$10^{-0.01}$	17:00	$0.66 = 0.69$	$10^{-0.79}$
Sleep onset	23:40	$0.46 < 0.50$	$10^{-2.15}$	23:30	$0.33 < 0.40$	$< 10^{-S}$	23:40	$0.31 < 0.39$	$10^{-6.00}$	00:10	$0.59 < 0.66$	$< 10^{-S}$
Onset to offset	07h10m	$0.48 > 0.42$	$10^{-3.72}$	08h20m	$0.52 > 0.47$	$< 10^{-S}$	07h10m	$0.49 > 0.39$	$< 10^{-S}$	08h40m	$0.51 > 0.42$	$< 10^{-S}$
Sleep time	08h30m	$0.84 = 0.82$	$10^{-0.22}$	08h20m	$0.44 = 0.41$	$10^{-0.78}$	08h50m	$0.70 = 0.76$	$10^{-0.68}$	08h30m	$0.37 > 0.33$	$10^{-2.64}$
**United States**												
Sleep offset	06:20	$0.59 < 0.61$	$10^{-2.97}$	07:40	$0.63 = 0.64$	$10^{-1.10}$	07:30	$0.67 = 0.65$	$10^{-0.94}$	07:00	$0.40 > 0.38$	$10^{-4.49}$
Center of gravity	14:00	$0.42 < 0.44$	$10^{-6.30}$	14:30	$0.38 < 0.40$	$10^{-4.78}$	13:40	$0.18 = 0.19$	$10^{-0.65}$	13:10	$0.05 = 0.06$	$10^{-0.48}$
Sleep onset	22:00	$0.38 < 0.41$	$< 10^{-S}$	21:20	$0.19 < 0.22$	$< 10^{-S}$	23:00	$0.55 < 0.57$	$10^{-2.32}$	21:20	$0.20 < 0.23$	$< 10^{-S}$
Onset to offset	07h50m	$0.59 > 0.56$	$10^{-4.28}$	09h30m	$0.70 > 0.68$	$10^{-2.19}$	08h20m	$0.59 > 0.55$	$10^{-4.33}$	10h00m	$0.69 > 0.66$	$< 10^{-S}$
Sleep time	07h40m	$0.53 > 0.51$	$10^{-4.27}$	09h20m	$0.62 > 0.60$	$10^{-2.46}$	08h10m	$0.50 > 0.46$	$10^{-3.99}$	09h50m	$0.62 > 0.59$	$< 10^{-S}$

Table S.1 KS statistics for the sleep/wake cycle after a permutation test of size $M = 10^7$ compared seasonal deviations $\Delta K_{ks} \propto (P_s - P_w)$ to permuted deviations ΔK^j_{ks}. Every analysis displays the time at $\max |\Delta K_{ks}|$, the cumulative distributions P_s and P_w at that index and a binary relational operator which put forward the result of the test: $=$ if the null hypothesis $H_0 : P_s - P_w = 0$ sustains; $>$ if the hypothesis $H_0 : P_s - P_w \leq 0$ does not sustain; $<$ if the hypothesis $H_0 : P_s - P_w \geq 0$ does not sustain; and $\neq$ if none of the null hypotheses sustains. Next to them the p-value that support the result: the fraction of permutations that sustained the alternate H_1. Significance is taken at the standard level $\alpha = 5\,\% = 10^{-1.30}$. The sensitivity of the p-value is $M^{-1} = 10^{-S}$ with $S = 7$.

KS statistics	t_m	Group 1 Mon-Fri employees $P_s \leftrightarrow P_w$	p	t_m	Group 3 Sat-Sun non-employees $P_s \leftrightarrow P_w$	p
**Great Britain**						
Labor onset	07:40	$0.25 = 0.23$	$10^{-0.29}$	10:00	$0.67 > 0.60$	$10^{-1.83}$
Center of gravity	13:50	$0.77 = 0.79$	$10^{-0.13}$	11:00	$0.30 = 0.26$	$10^{-0.33}$
Labor offset	16:20	$0.34 = 0.36$	$10^{-0.33}$	18:40	$0.65 = 0.69$	$10^{-0.25}$
Onset to offset	06h30m	$0.20 = 0.22$	$10^{-0.19}$	04h50m	$0.32 < 0.41$	$10^{-2.57}$
Labor time	07h40m	$0.48 = 0.52$	$10^{-1.09}$	05h00m	$0.38 < 0.49$	$10^{-3.53}$
**France**						
Labor onset	08:20	$0.50 = 0.52$	$10^{-0.42}$	08:00	$0.28 > 0.21$	$10^{-2.57}$
Center of gravity	10:20	$0.10 = 0.09$	$10^{-0.11}$	14:10	$0.47 = 0.44$	$10^{-0.23}$
Labor offset	12:00	$0.07 = 0.05$	$10^{-0.55}$	22:30	$0.83 = 0.87$	$10^{-0.60}$
Onset to offset	08h50m	$0.51 = 0.49$	$10^{-0.38}$	05h50m	$0.45 < 0.54$	$10^{-3.37}$
Labor time	07h30m	$0.52 = 0.50$	$10^{-0.25}$	05h40m	$0.61 < 0.69$	$10^{-2.78}$
**Italy**						
Labor onset	07:50	$0.38 > 0.34$	$10^{-2.23}$	08:10	$0.53 = 0.49$	$10^{-1.10}$
Center of gravity	14:50	$0.81 = 0.83$	$10^{-0.14}$	10:30	$0.25 = 0.22$	$10^{-0.47}$
Labor offset	14:50	$0.21 = 0.20$	$10^{-0.14}$	15:00	$0.37 = 0.34$	$10^{-0.28}$
Onset to offset	09h40m	$0.56 = 0.58$	$10^{-0.47}$	11h40m	$0.81 = 0.84$	$10^{-0.53}$
Labor time	08h40m	$0.72 = 0.74$	$10^{-0.57}$	03h30m	$0.15 = 0.18$	$10^{-0.40}$
**Spain**						
Labor onset	07:30	$0.23 = 0.20$	$10^{-0.79}$	06:30	$0.18 = 0.12$	$10^{-0.53}$
Center of gravity	11:40	$0.34 = 0.31$	$10^{-0.56}$	11:00	$0.26 = 0.22$	$10^{-0.11}$
Labor offset	20:10	$0.73 = 0.76$	$10^{-0.62}$	20:30	$0.64 = 0.71$	$10^{-0.78}$
Onset to offset	11h40m	$0.81 = 0.84$	$10^{-0.79}$	11h40m	$0.73 = 0.79$	$10^{-0.58}$
Labor time	09h10m	$0.77 = 0.80$	$10^{-0.76}$	06h00m	$0.48 = 0.44$	$10^{-0.19}$
**United States**						
Labor onset	06:50	$0.28 = 0.27$	$10^{-0.97}$	07:50	$0.34 = 0.33$	$10^{-0.36}$
Center of gravity	12:30	$0.56 = 0.57$	$10^{-0.84}$	21:30	$0.91 = 0.92$	$10^{-0.05}$
Labor offset	21:30	$0.86 = 0.87$	$10^{-0.77}$	18:10	$0.55 = 0.56$	$10^{-0.34}$
Onset to offset	10h50m	$0.76 = 0.78$	$10^{-1.02}$	06h00m	$0.43 = 0.42$	$10^{-0.22}$
Labor time	09h40m	$0.78 = 0.79$	$10^{-0.63}$	02h10m	$0.30 = 0.30$	$10^{-0.09}$

Table S.2 Same as Table S.1 but for the labour cycle.

Average values	Group 1 Mon-Fri employees $E_s \leftrightarrow E_w$	p	Group 2 Mon-Fri non-employees $E_s \leftrightarrow E_w$	p	Group 3 Sat-Sun employees $E_s \leftrightarrow E_w$	p	Group 4 Sat-Sun non-employees $E_s \leftrightarrow E_w$	p
**Great Britain**								
Sleep offset	06:44=06:48	$10^{-0.89}$	07:38=07:34	$10^{-0.93}$	07:08=07:17	$10^{-1.04}$	08:09<08:17	$10^{-2.96}$
Center of gravity	15:06=15:03	$10^{-0.66}$	15:31=15:30	$10^{-0.27}$	15:38=15:47	$10^{-1.11}$	15:51<15:57	$10^{-3.80}$
Sleep onset	23:16=23:13	$10^{-0.48}$	23:20=23:20	$10^{-0.06}$	23:43=23:49	$10^{-0.47}$	23:24=23:28	$10^{-1.08}$
Offset to onset	07h28m=07h34m	$10^{-1.09}$	08h19m=08h13m	$10^{-0.63}$	07h25m=07h29m	$10^{-0.20}$	08h45m=08h49m	$10^{-0.57}$
Sleep time	07h40m=07h43m	$10^{-0.62}$	08h40m>08h33m	$10^{-1.69}$	07h56m=08h02m	$10^{-0.48}$	09h03m=09h08m	$10^{-0.97}$
$\|E_s - E_w\|$ min;ave;max	(3;4;6)min		(1;4;7)min		(4;7;10)min		(4;5;8)min	
SEM (average)	2 min		2 min		4 min		2 min	
**France**								
Sleep offset	06:41>06:37	$10^{-3.62}$	07:31>07:28	$< 10^{-S}$	07:15=07:28	$10^{-0.76}$	07:55<08:00	$10^{-4.40}$
Center of gravity	15:00>14:55	$10^{-3.00}$	15:25=15:21	$10^{-0.37}$	15:41=15:39	$10^{-0.30}$	15:50=15:48	$10^{-0.47}$
Sleep onset	23:17=23:12	$10^{-0.54}$	23:17>23:13	$< 10^{-S}$	23:56>23:46	$10^{-5.33}$	23:38>23:33	$< 10^{-S}$
Offset to onset	07h24m=07h24m	$10^{-0.05}$	08h14m=08h15m	$10^{-1.01}$	07h19m<07h42m	$10^{-1.97}$	08h17m<08h27m	$< 10^{-S}$
Sleep time	07h35m=07h35m	$10^{-0.08}$	08h29m=08h31m	$10^{-0.40}$	07h52m<08h06m	$10^{-2.27}$	08h42m<08h49m	$10^{-2.55}$
$\|E_s - E_w\|$ min;ave;max	(0;3;5)min		(2;3;4)min		(2;12;24)min		(2;6;10)min	
SEM (average)	1 min		2 min		3 min		2 min	
**Italy**								
Sleep offset	06:45=06:47	$10^{-0.74}$	07:30=07:30	$10^{-0.09}$	06:58=07:00	$10^{-0.37}$	08:00<08:05	$10^{-3.24}$
Center of gravity	15:00=14:58	$10^{-0.57}$	15:21>15:17	$10^{-2.59}$	15:20=15:21	$10^{-0.17}$	15:45=15:44	$10^{-0.27}$
Sleep onset	23:10>23:06	$10^{-1.80}$	23:08>22:58	$10^{-6.52}$	23:37=23:34	$10^{-0.60}$	23:20>23:14	$10^{-5.54}$
Offset to onset	07h35m<07h41m	$10^{-2.52}$	08h22m<08h32m	$10^{-4.32}$	07h21m=07h27m	$10^{-0.89}$	08h40m<08h51m	$< 10^{-S}$
Sleep time	07h39m=07h42m	$10^{-0.74}$	08h34m<08h42m	$10^{-2.70}$	07h38m=07h44m	$10^{-0.97}$	08h53m<09h03m	$< 10^{-S}$
$\|E_s - E_w\|$ min;ave;max	(2;4;7)min		(0;6;10)min		(1;4;6)min		(1;6;11)min	
SEM (average)	1 min		1 min		2 min		1 min	
**Spain**								
Sleep offset	06:59<07:05	$10^{-1.61}$	08:14=08:16	$10^{-0.21}$	07:26<07:34	$< 10^{-S}$	08:44<08:53	$< 10^{-S}$
Center of gravity	15:31=15:33	$10^{-0.60}$	16:17>16:10	$10^{-5.20}$	16:11=16:11	$10^{-0.01}$	16:40=16:39	$10^{-0.26}$
Sleep onset	23:55>23:49	$10^{-2.46}$	00:02>23:51	$< 10^{-S}$	00:31>00:18	$< 10^{-S}$	00:11>00:00	$< 10^{-S}$
Offset to onset	07h05m<07h16m	$10^{-2.67}$	08h12m<08h25m	$10^{-3.36}$	06h54m<07h16m	$< 10^{-S}$	08h34m<08h53m	$< 10^{-S}$
Sleep time	07h29m=07h31m	$10^{-0.33}$	08h42m<08h48m	$10^{-1.63}$	07h56m=07h58m	$10^{-0.13}$	09h15m<09h24m	$10^{-2.48}$
$\|E_s - E_w\|$ min;ave;max	(2;5;11)min		(2;8;13)min		(0;9;21)min		(2;10;20)min	
SEM (average)	2 min		2 min		5 min		2 min	
**United States**								
Sleep offset	06:08>06:06	$10^{-2.14}$	07:16=07:14	$10^{-1.21}$	06:55=06:58	$10^{-0.81}$	07:43<07:47	$10^{-4.42}$
Center of gravity	14:30>14:26	$10^{-4.95}$	15:05>15:01	$10^{-3.76}$	15:12=15:11	$10^{-0.27}$	15:16=15:16	$10^{-0.07}$
Sleep onset	22:53>22:49	$10^{-3.65}$	22:49>22:45	$10^{-3.19}$	23:21>23:16	$10^{-2.13}$	22:46>22:43	$10^{-2.99}$
Offset to onset	07h15m=07h17m	$10^{-0.87}$	08h27m=08h29m	$10^{-0.57}$	07h34m<07h42m	$10^{-2.73}$	08h57m<09h04m	$10^{-7.00}$
Sleep time	07h39m<07h41m	$10^{-1.73}$	08h58m<09h03m	$10^{-2.70}$	08h16m<08h23m	$10^{-3.50}$	09h22m<09h30m	$< 10^{-S}$
$\|E_s - E_w\|$ min;ave;max	(2;3;4)min		(2;4;5)min		(1;5;8)min		(0;4;8)min	
SEM (average)	1 min		1 min		1 min		1 min	

Table S.3 Seasonal sample average values E_s and E_w for the stochastic variables related to the sleep/wake cycle. For every survey and group the minimum, average and maximum value of absolute differences $|E_s - E_w|$ and the average of the standard deviation of the averages (SEM) is listed. Between every E_s and E_w a binary relational operator puts forward the result of the permutation test with the p-value in the next column. The size of the permutation test was $M = 10^7$ ($S = 7$).

Average values	Group 1 Mon-Fri employees $E_s \leftrightarrow E_w$	p	Group 3 Sat-Sun employees $E_s \leftrightarrow E_w$	p
**Great Britain**				
Labor onset	08:50=08:57	$10^{-0.83}$	09:59<10:26	$10^{-1.60}$
Center of gravity	13:13=13:10	$10^{-0.25}$	13:54=13:50	$10^{-0.13}$
Labor offset	17:24=17:19	$10^{-0.36}$	17:19=16:56	$10^{-0.83}$
Onset of offset	08h34m=08h22m	$10^{-1.10}$	07h20m>06h29m	$10^{-3.22}$
Labor time	07h35m>07h25m	$10^{-1.67}$	06h08m>05h32m	$10^{-3.25}$
$\|E_s - E_w\|$ min;ave;max	(3;7;12)min		(5;28;51)min	
SEM (average)	4 min		10 min	
**France**				
Labor onset	09:00=08:58	$10^{-0.11}$	11:08<11:41	$10^{-2.32}$
Center of gravity	13:29=13:31	$10^{-0.10}$	14:52=14:49	$10^{-0.12}$
Labor offset	17:43=17:47	$10^{-0.19}$	18:04=17:43	$10^{-1.00}$
Onset of offset	08h43m=08h48m	$10^{-0.21}$	06h56m>06h02m	$10^{-3.83}$
Labor time	06h58m=07h01m	$10^{-0.41}$	04h44m>04h20m	$10^{-2.47}$
$\|E_s - E_w\|$ min;ave;max	(2;4;6)min		(4;27;54)min	
SEM (average)	3 min		8 min	
**Italy**				
Labor onset	08:24<08:32	$10^{-2.02}$	09:10=09:20	$10^{-0.77}$
Center of gravity	12:57=12:56	$10^{-0.09}$	13:11=13:20	$10^{-0.62}$
Labor offset	17:31=17:31	$10^{-0.02}$	17:13=17:16	$10^{-0.13}$
Onset of offset	09h07m=08h59m	$10^{-0.98}$	08h03m=07h56m	$10^{-0.37}$
Labor time	07h41m=07h36m	$10^{-0.79}$	06h40m=06h35m	$10^{-0.43}$
$\|E_s - E_w\|$ min;ave;max	(0;4;8)min		(3;7;10)min	
SEM (average)	3 min		5 min	
**Spain**				
Labor onset	08:48=08:56	$10^{-0.85}$	09:44=09:58	$10^{-0.20}$
Center of gravity	13:18=13:25	$10^{-0.81}$	14:06=14:06	$10^{-0.00}$
Labor offset	18:03=18:03	$10^{-0.01}$	17:53=17:55	$10^{-0.05}$
Onset of offset	09h15m=09h07m	$10^{-0.52}$	08h08m=07h57m	$10^{-0.13}$
Labor time	07h46m=07h42m	$10^{-0.44}$	06h28m=06h22m	$10^{-0.17}$
$\|E_s - E_w\|$ min;ave;max	(0;6;8)min		(0;7;14)min	
SEM (average)	4 min		11 min	
**United States**				
Labor onset	08:14=08:14	$10^{-0.05}$	10:54=10:52	$10^{-0.11}$
Center of gravity	12:53=12:49	$10^{-1.23}$	14:26=14:24	$10^{-0.14}$
Labor offset	17:37=17:33	$10^{-1.15}$	17:44=17:42	$10^{-0.19}$
Onset of offset	09h23m=09h19m	$10^{-0.91}$	06h50m=06h49m	$10^{-0.06}$
Labor time	08h01m=07h59m	$10^{-0.72}$	05h27m=05h23m	$10^{-0.64}$
$\|E_s - E_w\|$ min;ave;max	(0;3;4)min		(1;2;5)min	
SEM (average)	1 min		3 min	

Table S.4 Same as Table S.3 but for the labour variables.

Quartile differences	Group 1 Mon-Fri employees			Group 2 Mon-Fri non-employees			Group 3 Sat-Sun employees			Group 4 Sat-Sun non-employees		
	ΔQ_1	ΔQ_2	ΔQ_3	ΔQ_1	ΔQ_2	ΔQ_3	ΔQ_1	ΔQ_2	ΔQ_3	ΔQ_1	ΔQ_2	ΔQ_3
**Great Britain**												
Sleep offset, t_1						$+2$	-1		-2	-1	-1	-1
Center of gravity, t_2							-1	-1				
Sleep onset, t_3		$+1$				-1	-1	-1	-1			-1
Onset to offset, d_2	-1			$+1$		$+1$			-1	-1	-1	-1
Sleep time, d_1		-1			$+1$	$+2$			-1	-1		
**France**												
Sleep offset, t_1				-1		$+1$	-3	-1				
Center of gravity, t_2	$+1$	$+1$	$+1$						$+1$	$+1$		$+1$
Sleep onset, t_3							$+1$	$+2$	$+3$	$+1$	$+1$	$+1$
Onset to offset, d_2	-1	-1					-3	-1	-1	-1	-1	-1
Sleep time, d_1			-1				-1	-1	-1	-1	-1	-1
**Italy**												
Sleep offset, t_1												
Center of gravity, t_2			$+1$		$+1$							
Sleep onset, t_3			$+1$	$+2$	$+1$	$+1$			$+1$	$+1$	$+1$	
Onset to offset, d_2		-1	-1	-1	-2	-1	-1	-1	-1	-1	-2	-2
Sleep time, d_1		-1	-1	-2		-1	-1	-1	-1	-1	-1	
**Spain**												
Sleep offset, t_1	-1				-1		-3					-1
Center of gravity, t_2		-1			$+1$	$+1$					$+1$	$+1$
Sleep onset, t_3	$+1$	$+1$		$+2$		$+1$		$+2$	$+3$	$+1$		$+1$
Onset to offset, d_2		-1		-1	-1	-2	-2	-2		-2	-3	-2
Sleep time, d_1			-1				-2		$+2$	-1	-1	-1
**United States**												
Sleep offset, t_1												
Center of gravity, t_2	$+1$	$+1$				$+1$	$+1$					
Sleep onset, t_3	$+1$	$+1$	$+1$	$+1$		$+1$				$+1$	$+1$	
Onset to offset, d_2			-1			-1	-2		-2	-1		-2
Sleep time, d_1	-1	-1		-1	-1	-1		-2	-1		-2	-2

Table S.5 Seasonal differences in quartiles $\Delta Q = Q_s - Q_w$ of the sleep/wake cycle. Values display units of ten minutes —the discretization level of time use surveys—. Voids highlight null difference.

Quartile differences	Group 1 Mon-Fri employees			Group 2 Sat-Sun employees		
	ΔQ_1	ΔQ_2	ΔQ_3	ΔQ_1	ΔQ_2	ΔQ_3
**Great Britain**						
Labor onset, t_1	-1			-1	-2	-8
Center of gravity, t_2			$+1$	-2		$+2$
Labor offset, t_3				$+2$		$+3$
Onset to offset, d_2	$+1$			$+7$	$+5$	$+2$
Labor time, d_1	$+2$	$+1$	$+1$	$+5$	$+6$	$+1$
**France**						
Labor onset, t_1	-1	$+1$		-3	-2	-6
Center of gravity, t_2					-1	
Labor offset, t_3			-1	$+3$	$+1$	$+3$
Onset to offset, d_2		-1			$+10$	$+5$
Labor time, d_1	-1		-1		$+3$	$+4$
**Italy**						
Labor onset, t_1	-1		-1		-1	-1
Center of gravity, t_2				-1	-1	-1
Labor offset, t_3			-1	-1		
Onset to offset, d_2			$+1$	$+1$		$+1$
Labor time, d_1				$+2$	-1	
**Spain**						
Labor onset, t_1			-1	-1		-1
Center of gravity, t_2			-2	-1		$+2$
Labor offset, t_3			$+1$		-3	$+3$
Onset to offset, d_2			$+2$		-1	$+5$
Labor time, d_1				$+1$	-1	
**United States**						
Labor onset, t_1					-1	
Center of gravity, t_2			$+1$			$+1$
Labor offset, t_3			$+1$		$+1$	
Onset to offset, d_2	$+1$	$+1$	$+1$		-1	
Labor time, d_1			$+1$	$+1$	$+1$	

Table S.6 Same as Table S.5 but for the labour statistics.
